# Recent Discoveries in the Androgen Receptor Pathway in Castration-Resistant Prostate Cancer

**DOI:** 10.3389/fonc.2020.581515

**Published:** 2020-10-08

**Authors:** Daisuke Obinata, Mitchell G. Lawrence, Kenichi Takayama, Nicholas Choo, Gail P. Risbridger, Satoru Takahashi, Satoshi Inoue

**Affiliations:** ^1^Department of Urology, Nihon University School of Medicine, Tokyo, Japan; ^2^Monash Biomedicine Discovery Institute Cancer Program, Prostate Cancer Research Group, Department of Anatomy and Developmental Biology, Monash University, Clayton, VIC, Australia; ^3^Cancer Research Division, Peter MacCallum Cancer Centre, Melbourne, VIC, Australia; ^4^Sir Peter MacCallum Department of Oncology, The University of Melbourne, Parkville, VIC, Australia; ^5^Department of Systems Aging Science and Medicine, Tokyo Metropolitan Institute of Gerontology, Tokyo, Japan; ^6^Research Center for Genomic Medicine, Saitama Medical University, Saitama, Japan

**Keywords:** androgen receptor, castration-resistant prostate cancer, transcription factors, octamer transcription factor 1, preclinical models

## Abstract

The androgen receptor (AR) is the main therapeutic target in advanced prostate cancer, because it regulates the growth and progression of prostate cancer cells. Patients may undergo multiple lines of AR-directed treatments, including androgen-deprivation therapy, AR signaling inhibitors (abiraterone acetate, enzalutamide, apalutamide, or darolutamide), or combinations of these therapies. Yet, tumors inevitably develop resistance to the successive lines of treatment. The diverse mechanisms of resistance include reactivation of the AR and dysregulation of AR cofactors and collaborative transcription factors (TFs). Further elucidating the nexus between the AR and collaborative TFs may reveal new strategies targeting the AR directly or indirectly, such as targeting BET proteins or OCT1. However, appropriate preclinical models will be required to test the efficacy of these approaches. Fortunately, an increasing variety of patient-derived models, such as xenografts and organoids, are being developed for discovery-based research and preclinical drug screening. Here we review the mechanisms of drug resistance in the AR signaling pathway, the intersection with collaborative TFs, and the use of patient-derived models for novel drug discovery.

## Introduction

Prostate cancer is one of the most common causes of cancer-related death among men in Western countries. At diagnosis, most prostate cancers rely on the androgen receptor (AR) signaling for growth and survival. In this pathway, the AR is bound by ligands, such as dihydrotestosterone (DHT), and regulates the expression of target genes (1–5). In addition, the AR collaborates with cofactors, including transcription factors (TFs), which bind to specific DNA elements in regulatory regions of AR-responsive genes. Since AR collaborative TFs fine-tune androgen-responsive gene expression, it is important to further elucidate their role in the progression of prostate cancer.

In normal prostate epithelium, the AR suppresses proliferation and promotes differentiation (6); however, during carcinogenesis prostate cancer cells develop “lineage-addiction,” where the AR promotes tumor progression (7). Given the importance of the AR pathway in prostate cancer, it is the target of most treatments for advanced disease. Androgen deprivation therapy (ADT), through surgical or pharmacological castration, is initially effective at reducing tumor burden. ADT is administered alone or in combination with chemotherapy or AR-signaling inhibitors (8). A subset of cancer cells withstand treatment and eventually develop into castration-resistant prostate cancer (CRPC), which proliferates despite castrate concentrations of circulating androgens. Since AR signaling persists in most cases of CRPC, patients receive further treatment with AR signaling inhibitors (abiraterone acetate, enzalutamide, apalutamide, and darolutamide) based on whether they have metastatic or non-metastatic disease (9–12). Yet, tumors inevitably acquire further resistance, often by reactivating AR signaling. Once patients fail an AR signaling inhibitor, further treatments include another AR-directed therapy, chemotherapy, or if there are genomic defects in homologous recombination repair genes, a PARP inhibitor (13, 14). However, as CRPC is ultimately lethal, there is an ongoing need for new treatments.

An important step in developing novel therapies is testing their effectiveness in preclinical models. Although there is a longstanding paucity of preclinical models of CRPC, larger collections of patient-derived models are providing new tools to validate and prioritize candidate treatments for clinical trials. In this review, we examine mechanisms of castration-resistance involving the AR and collaborating TFs, new strategies for targeting tumors with these features, and the use of different patient-derived models for testing these novel treatments.

## Mechanisms of Castration-Resistance Through Alterations of the AR

The AR gene on Xq11-13 consists of 8 exons encoding the N-terminal domain (NTD; 555 amino acids; exon 1), DNA-binding domain (DBD; 68 amino acids; exons 2 and 3), hinge region (40 amino acids; exon 4), and ligand binding domain (LBD; 295 amino acids; exons 4–8) (15, 16). Binding of androgens to the LBD triggers an intramolecular interaction with the NTD, which in turn interacts with AR co-activators (17, 18).

Amplifications of the *AR* locus are one the most common mechanisms of castration-resistance, and they often encompass an enhancer located ~700 kilobases upstream (19–21). In some tumors, the *AR* gene and enhancer are amplified independently of each other (21). The *AR* enhancer is bound by several transcriptional activators, including FOXA1, GATA2, NKX3.1, HOXB13, and the AR itself (20). Amplifications of the *AR* and its enhancer are associated with higher levels of AR expression, and over-expressing the AR in prostate cancer cell lines causes enzalutamide-resistance (20, 21). Accordingly, patients with amplifications of the *AR* locus and/or enhancer are often resistant to AR-directed therapies, including enzalutamide and abiraterone acetate (22, 23). In preclinical studies with VCaP cells, which have an *AR* amplification and express high levels of the *AR*, darolutamide had a lower IC50 than enzalutamide and apalutamide in suppressing proliferation (24), suggesting that potent inhibition of the AR may be required for tumors with this mechanism of resistance.

The conformation of the AR can be disrupted by point mutations, which commonly arise in CRPC and mediate resistance to AR-directed treatments (25, 26). Occasionally, two AR mutations can occur in the same tumor (27–30). Point mutations often occur in the LBD, causing gain-of-function in ligand binding, so the AR is activated by other steroids, and antagonists, like enzalutamide, are converted into agonists (31–33) (Supplementary Table 1). Since AR mutations confer resistance to particular antagonists, they are potential predictive biomarkers for AR-directed inhibitors. Enzalutamide may not be suitable for tumors with AR mutations that convert it into a partial agonist (F877L, H875Y/T878A, F877L/T878A) (27). Darolutamide might be more effective for these tumors, since it remains an antagonist despite these AR mutations (34). In addition, darolutamide has unique flexibility that allows it to bind the W742C/L mutated ligand-binding pocket, unlike enzalutamide (35). However, the utility of AR mutations as biomarkers needs confirmation in patients. For example, the F877L AR mutation converts apalutamide into a partial agonist *in vitro*, but neither this mutation nor T878A was a common cause of acquired resistance to apalutamide in a phase I/II trial (36).

**Figure 1:**
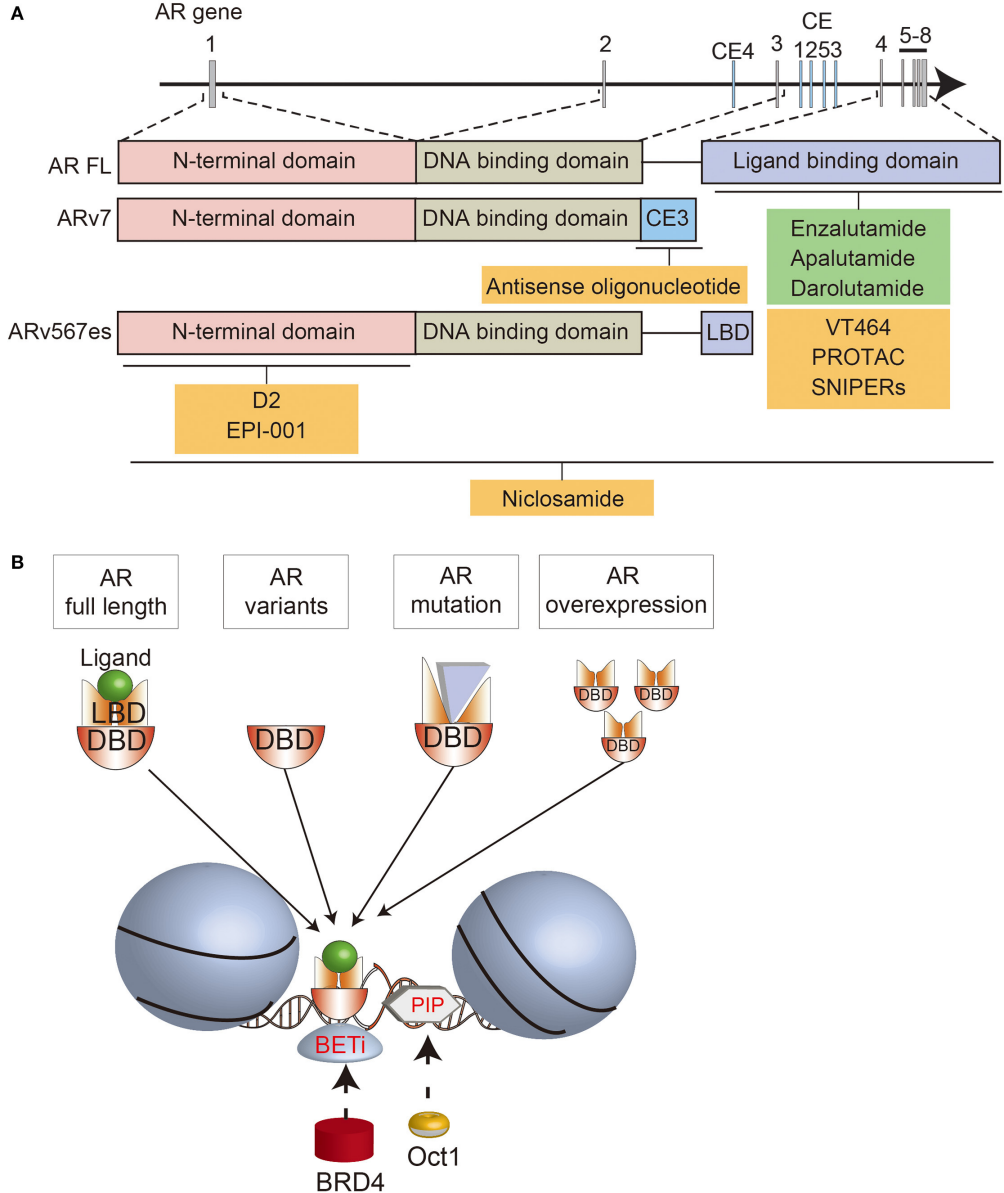
Schematic summary of the AR structure, AR-directed treatments, and interactions with BRD4 and OCT1. **(A)** Overview of the AR locus, the structure of full length (FL) and variant (AR-V7, ARv567es) forms of the AR, and various AR-targeted treatments that are approved (green) or in development (yellow). **(B)** Summary of the interactions between different forms of the AR, BRD4, and OCT1 on chromatin.

In addition to AR mutations, constitutively active AR splice variants (ARVs) can mediate castration resistance (37). Increased expression of ARVs can arise through amplifications or structural rearrangements of the AR gene in CRPC (20, 30, 38, 39). Among numerous ARVs, AR-V7, and ARv567es have been studied in the most detail. AR-V7 includes exons 1/2/3, encoding the NTD, followed by a cryptic exon (Figures 1A,B) (37). ARv567es includes exons 1/2/3/4/8, but skips exons 5/6/7 (40). Since both variants lack the LBD, they are not bound by most AR signaling inhibitors, so can sustain AR-driven gene expression. The lack of the AR hinge region in AR-V7 may also promote therapy resistance. SPOP (speckle type POZ protein), an E3 ubiquitin ligase that is upregulated by enzalutamide treatment, usually binds to the hinge region of the AR and induces its degradation (41). By escaping this ubiquitin degradation pathway, AR-V7 may enable enzalutamide resistance. In addition, the hinge region usually mediates microtubule binding and translocation of the AR into the nucleus (42, 43). Since AR-V7 lacks the hinge region, its transport is independent of microtubules, enabling resistance to taxane chemotherapy, which targets microtubules, unlike ARv567es which still contains the hinge region (43, 44). Cell-cycle or cell-division associated genes such as ubiquitin-conjugating enzyme E2 C (UBE2C) are unique AR-V7 targets, contributing to cell proliferation under androgen-depleted conditions (45, 46). Recent functional analyses demonstrated the importance of various splicing factors, which are highly expressed in CRPC tissues (47–50). Enhanced expression of splicing factors would promote their recruitment to pre-mRNA, facilitating the mRNA splicing process. Thus, altered splicing machinery would result in a dysregulated AR splicing process. Splicing factor, proline- and glutamine-rich (PSF/SFPQ) is responsible for wide-ranging upregulation of spliceosome gene expression in CRPC to activate a broad range of oncogenic pathways, including AR (48). Thus, these studies provide an intriguing insight into prostate cancer progression through splicing machinery.

## New Strategies for Directly Targeting the AR in CRPC

In an effort to overcome resistance to current treatments, new therapies are being developed to target the AR. Some compounds have a similar mechanisms-of-action to existing AR-directed treatments. Like abiraterone actetate, the new compound VT464 (seviteronel) is a CYP17A1 inhibitor that suppresses androgen biosynthesis (51). Unlike abiraterone acetate, VT464, selectively inhibits the 17,20-lyase rather than 17α-hydroxylase reactions, so it is proposed that the combination with prednisone is not necessary. However, phase 1 testing of VT464 suggested that there is minor inhibition of CYP17 hydroxylase (52), so low-dose dexamethasone is being administered with VT464 in ongoing trials with prostate cancer patients (53, 54). Abiraterone acetate and VT464 both also function as competitive AR antagonists, including of AR mutants, with VT464 more potent than abiraterone in cells with the T878A AR mutation (55–58).

An alternative strategy is to deplete the AR in prostate cancer cells. This may be done by blocking gene expression with antisense oligonucleotides targeting different regions of *AR* pre-mRNA transcripts (59–62). Antisense oligonucleotides against exon 1 reduce full-length AR and ARV expression, while antisense oligonucleotides against cryptic splicing signals specifically downregulate AR-V7 expression (60, 62). The AR can also be depleted using selective AR degraders (SARD), which bind to the AR and induce proteasome-mediated degradation (63). Some SARDs bind to both the N- and C-termini of the AR, so also promote degradation of ARVs (64). Preclinical studies suggested that niclosamide, an approved treatment for parasitic worms, could be repurposed as a SARD, since one of its effects is degradation of ARVs. Although the combination of niclosamide with enzalutamide or abiraterone significantly reduced the growth of castrate-resistant cells (65–67), a phase I trial showed that inhibitory concentrations of niclosamide could not be achieved in patients (68). Therefore, this approach must rely on newer generations of SARDs being developed (64).

Another way of inducing AR degradation is with proteolysis targeting chimeras (PROTAC) or SNIPERs (specific and non-genetic inhibitor of apoptosis protein [IAP]-dependent protein erasers). These heterobifunctional small molecules contain a ligand that binds to the target protein, such as an AR antagonist, linked to another ligand that engages the ubiquitin ligase complex (69–71). Since current AR-targeted PROTACs bind to the LBD, they induce degradation of full-length AR, but not ARVs. Nevertheless, they still inhibit the growth of enzalutamide-resistant cells, emphasizing the ongoing importance of full-length AR in many cases of CRPC (71).

A different strategy for directly targeting the AR is to disrupt its interactions with other molecules. D2 is a peptidomimetic that disrupts the interaction between the AR and a co-regulator, PELP1 (proline, glutamate and leucine rich protein 1), by mimicking the LXXLL motif in the AF2 domain of the AR C-terminus (72). By blocking this interaction, D2 inhibits nuclear translocation of the AR and reduces the growth of prostate cancer cells. EPI-001 also blocks the interactions between the AR and coactivators, and is notable because it binds the NTD (73). Thus, EPI-001 also inhibits ARVs (74). EPI-001 inhibits the growth of prostate cancer cell lines *in vitro* and *in vivo*, and had increased activity in combination with docetaxel (74, 75). However, off-target effects have been identified, highlighting the difficulty of targeting the AR NTD (76). EPI-506 was developed as a successor to EPI-001 (77), but was required at high doses in a phase I trial due to low potency and a short half-life. Therefore, the development of N-terminal AR inhibitors is ongoing (78).

Compounds are also being developed to block the interaction of the AR with DNA. This could target both full-length and variant forms of the AR, which contain the DBD. For example, AR binding to specific androgen response element sequences can be blocked using PI polyamides, N-methylimidazole (Im) and N-methylpyrrole (Py) amino acids that bind to the minor groove of DNA with high affinity and sequence specificity (79–81). PI polyamides that bind particular AREs can suppress androgen-responsive gene expression (82), and inhibit binding of RNA polymerase II to the transcription start site of AR-driving genes (83).

## Indirectly Targeting AR Signaling via Cofactors and Collaborating Transcription Factors

Cofactors, including coregulators and TFs, are also necessary for AR-regulated gene expression. Whilst coregulators directly bind to activation function (AF) 1 or 2 domains of the AR, TFs bind to DNA elements near AR binding sites (84). Some TFs are also pioneer factors that facilitate AR recruitment to target regions through chromatin remodeling (85). Dysregulation of TFs can dramatically change the pattern of AR responsive gene expression. Indeed, there are differences in AR binding regions and coordinating TFs between treatment-naïve and castration-resistant prostate cancer (86). AR binding sites that are unique to CRPC were not AR-regulated in treatment-naïve prostate cancer cells or enriched in binding of common AR collaborative TFs, such as MYC (86). MYC is a oncogenic transcription factor that plays a critical role in prostate cancer progression by influencing diverse molecular mechanisms (87).

The importance of cofactors and collaborative TFs makes them potential therapeutic targets for indirectly targeting the AR. There are numerous strategies for targeting different AR interacting proteins, so here we focus on two notable examples, bromodomain and extra terminal domain (BET) proteins and OCT1 (POU2F1; POU class 2 homeobox 1) that collaborate with MYC.

## BET Proteins

The BET family of epigenetic readers, including BRD2/3/4 (bromodomain containing 2, 3, and 4) and BRDT (bromodomain testis associated), bind to acetylated histones and regulate the expression of downstream genes such as MYC (88).

BET proteins are therapeutic targets in different tumor types, but are of particular interest in prostate cancer because they affect the expression and activity of the AR pathway (89). BET proteins directly interact with the NTD of the AR (90). Moreover, BRD4 has numerous shared DNA binding loci with full-length AR and AR-V7 (90, 91). With FOXA1, BRD4 and AR-V7 bind to canonical AR target genes, but with ZFX they bind to non-canonical genes related to cell cycle, autophagy, and WNT signaling (91). Accordingly, BET inhibitors downregulate the expression of AR target genes, as well as MYC (90, 91). BET inhibitors also decrease AR-V7 levels by regulating alternative splicing (92, 93). This culminates in reduced growth of prostate cancer cell lines, organoids and xenografts treated with BET inhibitors, including enzalutamide-resistant models (90, 92, 94, 95).

The promising preclinical data for BET inhibitors suggests that they are potential new treatments for CRPC, functioning in part by indirectly targeting the AR. Numerous BET inhibitors are clinical development and some are in phase I/II clinical trials enrolling men with CRPC, such as ABBV-075 (mivebresib) and MK-8628/OTX015 (birabresib) (89). So far, prostate cancer patients in these trials have still had progressive or stable disease, although a partial response has been reported (89, 96, 97). Ongoing trials are also testing combination treatments of BET inhibitors with AR-directed treatments, PARP inhibitors, chemotherapy and immunotherapy (89, 98). For example, a phase 1b/2a trial of the BET inhibitor ZEN-3694 in combination with enzalutamide demonstrated that the treatment had acceptable tolerability in men with metastatic CRPC who had previously failed abiraterone or enzalutamide (99). Encouragingly, a subset of these patients had prolonged progression-free survival with the combination therapy, including those with tumors with low AR activity.

A challenge in the clinical development of BET inhibitors is overcoming toxicity and off-target effects, so new forms of BET inhibitors are being developed. Using the PROTAC approach, BET degraders target BET proteins for ubiquitination and proteasomal destruction (71, 100). In addition, compounds have been developed to selectively target one of the two bromodomains (BD1 and BD2) within BET proteins (101, 102). BET degraders and selective bromodomain inhibitors both inhibit the growth of prostate cancer cells *in vitro* and *in vivo* (71, 100, 101). Therefore, ongoing trials, combination treatments and new compounds, may provide opportunities to treat CRPC by targeting BET proteins.

## OCT1

Another canonical AR collaborative TF is OCT1. Of the eight OCT proteins, OCT1 is most widely expressed, and is related to the pluripotency master regulator OCT4 (103, 104). OCT1 acts downstream of pioneer factors that make histone modifications to support AR binding to target regions. GATA2 (GATA binding protein 2) and OCT1 work in a hierarchical network where GATA2 is recruited with AR, followed by OCT1 binding to its motifs. Increased immunoreactivity of OCT1 is correlated with worse prognosis of localized prostate cancer (105). OCT1 is also highly expressed in other cancers, including gastric and colorectal cancer (106, 107). Interestingly, in MYC-driven lung adenocarcinoma, OCT1 binding sites were enriched in a set of genes regulated by MYC (108), suggesting that OCT1 and MYC may also co-regulate a subset of androgen responsive genes in prostate cancer. Furthermore, OCT1 interacts with PARP-1 and BRCA1 (109, 110). OCT1 enhances breast cancer aggressiveness, and BRCA1 catalyzes OCT1 degradation to inhibit tumorigenicity (110). PARP inhibitors are often effective for cancers with *BRCA1* mutations, however some tumors are resistant (111). These findings suggest that OCT1 may have a significant effect when used in combination with PARP inhibitors.

Of the genes that are jointly regulated by OCT1 and the AR in prostate cancer, acyl-CoA synthetase long-chain family member 3 (ACSL3) is the mostly highly differentially expressed (112). ACSL3 in turn increases AKR1C3 (aldo-keto reductase family 1 member C3) expression, enhancing the backdoor pathway of androgen synthesis that confers resistance to abiraterone (113, 114). Beyond ACSL3, the genome-wide network of OCT1 target genes in CRPC is enriched in factors such as anillin actin binding protein (ANLN) and DLG associated protein 5 (DLGAP5) that regulate proliferation and migration (115, 116).

Although there are few drugs targeting OCT1, PI polyamides have been developed that block the interaction between OCT1 and specific DNA binding sites. A PI polyamide targeting the OCT1 binding sites of ACSL3 suppresses ACSL3 expression and inhibits the growth of CRPC by repressing global OCT1 chromatin association and AR signaling (112). These preclinical data support further development of compounds targeting OCT1 in CRPC.

## Patient-Derived Models for Testing New Treatments for CRPC

As novel compounds are developed to directly and indirectly target the AR, their efficacy must be tested with appropriate preclinical models. Unfortunately, the development of preclinical models of CRPC lags behind the evolving understanding of CRPC and changes in clinical practice. Most studies use a small collection of cell lines, including LNCaP, PC3, DU145, VCaP, 22RV1, and LAPC4 cells (117). These cells are very well-characterized and have been used for important discoveries. They have different mechanisms of castration-resistance, such AR amplification and expression of AR-V7 in VCaP cells, and an intragenic duplication of the *AR* gene and expression of several AR isoforms in 22Rv1 cells (40, 47, 118, 119). An important use of prostate cancer cell lines is high-throughput screening, including in the NCI-60 panel (120, 121). With this approach, cell lines can be used to identify drug targets with genome-wide genetic screens, such as with siRNA or CRISPR-Cas9, and treated with large compound libraries to identify candidate drugs for further evaluation (50, 122). Nevertheless, this small number of cell lines does not encompass the heterogeneity of CRPC. To address this challenge, there are ongoing efforts to develop new *in vivo, ex vivo*, and *in vitro* models from patient specimens.

The ability to establish patient-derived xenografts (PDXs) from patient tumors has advanced with the use of more highly immune-deficient strains of mice. Yet, PDXs are often more difficult to establish from prostate cancer compared to other malignancies, due to low take rates (10–40%) and long latency periods (up to 12 months) (123). Nevertheless, several groups have established collections of serially transplantable prostate cancer PDXs (30, 124–128). At least 51 PDXs of CRPC have been established, primarily from patients who failed ADT, but fewer from men treated with second generation AR-directed therapies (125). To simulate androgen deprivation, PDXs of CRPC are often grafted in castrated mice, with circulating androgen levels equivalent to patients treated with abiraterone (129). By increasing the number of models of CRPC, PDXs provide new opportunities to study the mechanisms of castration resistance, including mutations and ARVs. PDXs are also useful for testing whether candidate therapeutics are effective at reducing the growth of tumors with diverse alterations in the AR pathway. The typical endpoint to determine whether drug treatment reduces the growth rate of PDXs is decreased tumor volume, or ideally regression.

**Figure 2:**
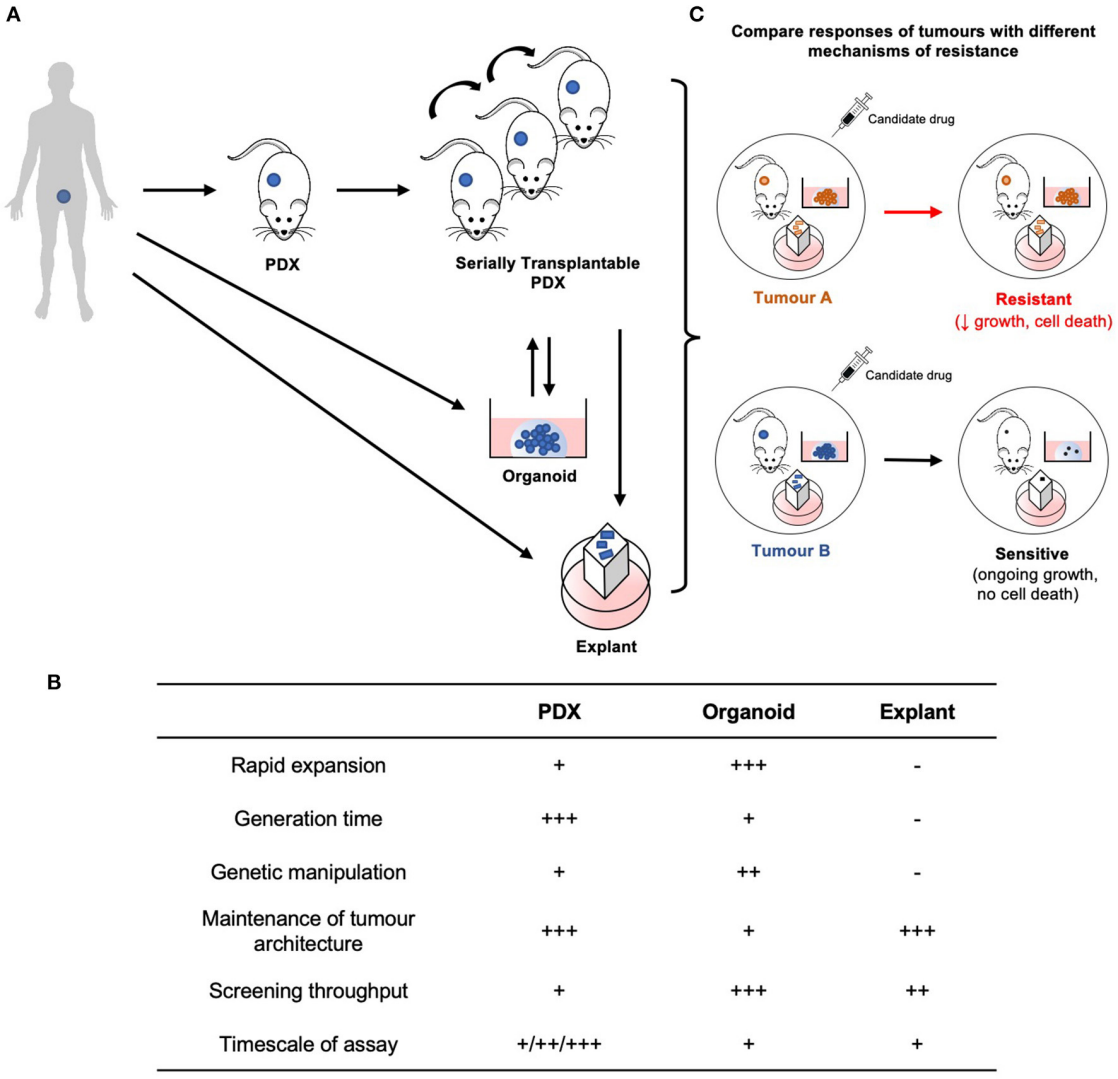
Establishment and application of patient-derived models for preclinical testing of new treatments for CRPC. **(A)** PDXs are established from human patient tumor tissue, and are considered serially transplantable when repassaged into additional host mice and expanded. Explants and organoids can be established directly from fresh patient specimens or from PDXs. PDXs may also be established from organoids. **(B)** Each model is unique with its own advantages and limitations. **(C)** Therefore, by integrating these models established from tumors with different resistance mechanisms, preclinical therapeutic evaluation can be performed with greater rigor and efficiency.

Like all experimental models, PDXs have limitations, so they can be integrated with other patient-derived models to maximize the advantages and offset the limitations of each approach (Figures 2A–C). PDXs provide a rigorous way to evaluate *in vivo* drug responses, but the experiments are expensive, labor-intensive, low throughput and have long timeframes. Explants and organoids are complementary models that address these limitations. Explants are intact pieces of tissue maintained for several days *ex vivo* on filters or gelatin sponges, so they retain the native tissue architecture and microenvironment (130, 131). Organoids are digested prostate tissue grown in extracellular matrix solutions, such as Matrigel. Explants and organoids can be established from fresh patient specimens or PDXs, which are renewable sources of tissue (30, 132, 133). These patient-derived models are higher throughput and can be used to rapidly test whether compounds affect proliferation and apoptosis. These *ex vivo* and *in vitro* cultures are also useful for testing tool compounds that have poor bioavailability or are not available in sufficient quantities for *in vivo* experiments. Explants and organoids can also be used for experiments that are challenging with PDXs, including large-scale dose responses of single or combination treatments, genetic manipulation, and short-term time points for mechanistic studies (132, 134, 135). This bridges the gap between high-throughput experiments with cell lines, and *in vivo* treatments with PDXs. Therefore, by combining different patient-derived models established from different cases of CRPC, it will be possible to test the next generation of therapies with greater rigor and efficiency to help prioritize them for further clinical trials.

## Conclusion

Over the last decade, the introduction of new treatments for CRPC has extended patient survival, but tumors still eventually fail treatment. The increasingly detailed understanding of the underlying mechanisms of resistance has facilitated the development of novel compounds that use alternative approaches to target the AR pathway, directly or indirectly. Two examples of drug targets are BET proteins, with BET inhibitors in ongoing clinical trials for prostate cancer, and OCT1, with novel compounds in preclinical development. Whether these compounds are effective as monotherapies, or should be used in combination with other treatments is still under investigation. Nevertheless, growing collections of patient-derived models, spanning xenografts, organoids and explants, are providing ways to test the efficacy of these candidate drugs across a wider spectrum of tumors. Collectively, this ongoing effort will provide a rich pipeline of new treatments for further validation in clinical trials.

## Author Contributions

DO, ST, and SI: conceived the concepts. DO, ML, and NC: wrote the first draft of the manuscript. KT and GR: revise the first draft. All authors reviewed and approved of the final manuscript.

## Conflict of Interest

The authors declare that the research was conducted in the absence of any commercial or financial relationships that could be construed as a potential conflict of interest.
