# Supplementary material for: Recent Discoveries in the Androgen Receptor Pathway in Castration-Resistant Prostate Cancer
Source: Front Oncol. 2020 Oct 8;10:581515. doi: 10.3389/fonc.2020.581515 (PMC7578370; doi:10.3389/fonc.2020.581515)
Supplement: Supplementary file 1 [file Table_1.DOCX]

Supporting Table: Summary of prominent AR mutations and their effects on AR activity.

AR mutation/s Affected agents Effect Ref

T878A/S Abiraterone/ Enzalutamide Progesterone agonistic/ agonistic (1, 2)

C687Y / T878A Enzalutamide/ Dexamethozone Agonistic (3)

F877L Enzalutamide/ Apalutamide Agonistic (4, 5)

F877L / T878A Enzalutamide Agonistic (6)

M896V / S889G Enzalutamide Agonistic (2)

W742L Enzalutamide / Apalutamide Agonistic (1)

H875Y Abiraterone Progesterone agonistic (7)

L702H Dexamethozone Agonistic (8)

1. Chen EJ, Sowalsky AG, Gao S, Cai C, Voznesensky O, Schaefer R, et al. Abiraterone treatment in castration-resistant prostate cancer selects for progesterone responsive mutant androgen receptors. Clin Cancer Res. 2015;21(6):1273-80.

2. Lallous N, Volik SV, Awrey S, Leblanc E, Tse R, Murillo J, et al. Functional analysis of androgen receptor mutations that confer anti-androgen resistance identified in circulating cell-free DNA from prostate cancer patients. Genome Biol. 2016;17:10.

3. Lawrence MG, Obinata D, Sandhu S, Selth LA, Wong SQ, Porter LH, et al. Patient-derived Models of Abiraterone- and Enzalutamide-resistant Prostate Cancer Reveal Sensitivity to Ribosome-directed Therapy. Eur Urol. 2018;74(5):562-72.

4. Balbas MD, Evans MJ, Hosfield DJ, Wongvipat J, Arora VK, Watson PA, et al. Overcoming mutation-based resistance to antiandrogens with rational drug design. Elife. 2013;2:e00499.

5. Fizazi K, Smith MR, Tombal B. Clinical Development of Darolutamide: A Novel Androgen Receptor Antagonist for the Treatment of Prostate Cancer. Clin Genitourin Cancer. 2018;16(5):332-40.

6. Prekovic S, van Royen ME, Voet AR, Geverts B, Houtman R, Melchers D, et al. The Effect of F877L and T878A Mutations on Androgen Receptor Response to Enzalutamide. Mol Cancer Ther. 2016;15(7):1702-12.

7. Duff J, McEwan IJ. Mutation of histidine 874 in the androgen receptor ligand-binding domain leads to promiscuous ligand activation and altered p160 coactivator interactions. Mol Endocrinol. 2005;19(12):2943-54.

8. Romanel A, Gasi Tandefelt D, Conteduca V, Jayaram A, Casiraghi N, Wetterskog D, et al. Plasma AR and abiraterone-resistant prostate cancer. Sci Transl Med. 2015;7(312):312re10.
